# Identical, but not the same: Intra-site and inter-site reproducibility of fractional anisotropy measures on two 3.0 T scanners

**DOI:** 10.1016/j.neuroimage.2010.03.046

**Published:** 2010-07-15

**Authors:** Christian Vollmar, Jonathan O'Muircheartaigh, Gareth J. Barker, Mark R. Symms, Pamela Thompson, Veena Kumari, John S. Duncan, Mark P. Richardson, Matthias J. Koepp

**Affiliations:** a National Society for Epilepsy MRI Unit, Dept. of Clinical and Experimental Epilepsy, UCL Institute of Neurology, Queen Square London WC1N 3BG, UK; b King's College London, Institute of Psychiatry, Dept. of Clinical Neuroscience, De Crespigny Park, London SE5 8AF, UK

## Abstract

Diffusion Tensor Imaging (DTI) is being increasingly used to assess white matter integrity and it is therefore paramount to address the test–retest reliability of DTI measures. In this study we assessed inter- and intra-site reproducibility of two nominally identical 3 T scanners at different sites in nine healthy controls using a DTI protocol representative of typical current “best practice” including cardiac gating, a multichannel head coil, parallel imaging and optimized diffusion gradient parameters. We calculated coefficients of variation (CV) and intraclass correlation coefficients (ICC) of fractional anisotropy (FA) measures for the whole brain, for three regions of interest (ROI) and for three tracts derived from these ROI by probabilistic tracking. We assessed the impact of affine, nonlinear and template based methods for spatially aligning FA maps on the reproducibility. The intra-site CV for FA ranged from 0.8% to 3.0% with ICC from 0.90 to 0.99, while the inter-site CV ranged from 1.0% to 4.1% with ICC of 0.82 to 0.99. Nonlinear image coregistration improved reproducibility compared to affine coregistration. Normalization to template space reduced the between-subject variation, resulting in lower ICC values and indicating a possibly reduced sensitivity. CV from probabilistic tractography were about 50% higher than for the corresponding seed ROI.

Reproducibility maps of the whole scan volume showed a low variation of less than 5% in the major white matter tracts but higher variations of 10–15% in gray matter regions.

One of the two scanners showed better intra-site reproducibility, while the intra-site CV for both scanners was significantly better than inter-site CV. However, when using nonlinear coregistration of FA maps, the average inter-site CV was below 2%. There was a consistent inter-site bias, FA values on site 2 were 1.0–1.5% lower than on site 1. Correction for this bias with a global scaling factor reduced the inter-site CV to the range of intra-site CV. Our results are encouraging for multi-centre DTI studies in larger populations, but also illustrate the importance of the image processing pipeline for reproducibility.

## Introduction

Diffusion Tensor Imaging (DTI) is an advanced Magnetic Resonance Imaging (MRI) technique that allows the assessment of water diffusion in the brain. In highly organized tissue like cerebral white matter, diffusion preferentially follows the longitudinal direction of axonal bundles and myelin sheaths while transverse diffusivity is limited by cell membranes, organelles and other structures. The degree of this directionality is described by the fractional anisotropy (FA) and high FA values represent highly anisotropic diffusion. FA is commonly used as a measure of white matter organization or white matter integrity, being higher in densely packed, parallel white matter bundles such as the corpus callosum (CC). FA measures are increasingly used in clinical studies and have shown alterations in various brain diseases such as multiple sclerosis (Ge et al., 2005) and epilepsy (Focke et al., 2008), as well as in normal aging (Sullivan and Pfefferbaum, 2006).

The intra-site test–retest reliability of DTI measures has been addressed mainly at 1.5 Tesla (T) (Ciccarelli et al., 2003; Pfefferbaum et al., 2003; Heiervang et al., 2006; Bonekamp et al., 2007) with just two recent studies at 3 T (Jansen et al., 2007; Bisdas et al., 2008) (Table 1). There is considerably less data on *cross centre* reliability of DTI measures; previous studies have shown large variability of FA quantification on different 1.5 T scanners (Cercignani et al., 2003; Pfefferbaum et al., 2003) with an expected higher inter-site than intra-site variability (Pfefferbaum et al., 2003).Typical current “best practice” 3 T DTI protocols differ considerably from older 1.5 T versions, with the inclusion of modern array head coils resulting in higher signal to noise ratios, and the increasing use of parallel imaging methods. There is little or no information on the inter-site reproducibility of measurements made using these recent MR technological developments. Reproducibility studies require image coregistration, for which there are several possible methods, such as affine, nonlinear and template based approaches. The quality of these coregistration procedures is likely to affect measurement reproducibility. As repeat measurements of the same subject need to be coregistered, it appears to be the most straightforward approach to coregister repeat scans in each subject's native space, avoiding any additional image transformation. However in daily life, it is common practice to use nonlinear normalizations to a common template space before further analysis and we have therefore directly compared both approaches.

Clinical studies often target very specific patient populations which are difficult to recruit by one imaging centre alone. Large scale pharmacological investigations are usually multi-centre studies that increase statistical power by pooling patients, but differences in MRI scanner manufacturers, models and set-ups even for the same type of scanner restrict the comparison of imaging parameters across sites. A necessary first step is the acquisition of test–retest data in controls for the assessment of reliability. Test–retest studies allow for an estimation of reproducibility, i.e. within-subject differences.

The purpose of the current study is fourfold:

1. To assess the reproducibility of DTI measures using a contemporary 3 T high field scanner system and a protocol typical of that which might be used in multi-centre studies using a variety of scanners.
2. To determine whether using this protocol on two nominally identical GE Signa HDx scanners at different sites (National Society for Epilepsy MRI Unit and Institute of Psychiatry, King's College London) results in acceptably low levels of cross-site variability.
3. To assess the impact of different steps of the image processing pipeline on measurement reproducibility: we compared different methods for image coregistration, for ROI definition and the effect of tractography compared to ROI analysis of FA maps.
4. To assess the measurement reproducibility within the scan volume, creating reproducibility maps to identify regions of unfavorably high FA variability.

## Methods

### Subjects

Nine healthy subjects (2 female, age range 28–52 years) underwent four MRI scans each, two at each imaging site. The order of scans across sites was randomized, the interval between individual scans ranged from 1 to 95 days, and all scans were acquired within a 12 month period. The study was approved by the Research Ethics Committee of the UCL Institute of Neurology and UCL Hospitals and written informed consent was obtained from each participant.

### MR image acquisition

A 3 T MRI scanner was used at each site, with imaging gradients with a maximum strength of 40 mT/m and slew rate 150 mT/m/s (GE Signa HDx, General Electric, Milwaukee, WI, USA.). The body coil was used for RF transmission, and an 8 channel head coil for signal reception, allowing a parallel imaging (ASSET) speed up factor of two. Each volume was acquired using a multi-slice peripherally-gated doubly refocused spin echo EPI sequence, optimized for precise measurement of the diffusion tensor in parenchyma, from 60 contiguous near-axial slice locations with 128 × 128 isotropic (2.4 × 2.4 × 2.4 mm) voxels. The echo time was 104.5 ms while to minimize physiological noise, cardiac gated triggering with a peripheral pulse sensor was applied (Wheeler-Kingshott et al., 2002) and the effective repetition time varied between subjects in the range between 12 and 20 RR intervals. Based on the recommendations of Jones et al. (2002), the maximum diffusion weighting was 1300 s mm^− 2^, and at each slice location, 4 images were acquired with no diffusion gradients applied, together with 32 diffusion weighted images in which gradient directions were uniformly distributed in space. The total acquisition time for this sequence was approximately 10 min, depending on the heart rate.

### Image processing

Image distortions induced by eddy currents and subject movement during the acquisition were corrected using a mutual information based affine realignment of all volumes to the first non-diffusion weighted volume (FSL 4, http://www.fmrib.ox.ac.uk/fsl/) (Behrens et al., 2003). The brain tissue was automatically segmented from skull and background using FSL's deformable brain model based Brain Extraction Tool (Smith, 2002). Brain extraction was performed on a non-diffusion weighted volume with a fractional intensity threshold of 0.3 and then applied to the whole realigned DTI acquisition.

Diffusion tensors were reconstructed from the 32 diffusion weighted volumes using Camino software (http://www.cs.ucl.ac.uk/research/medic/camino/, Version 2, rev 530), (Cook et al., 2006). The resulting diffusion tensors were diagonalized, yielding the three principal eigenvalues *λ*_1_, *λ*_2_ and *λ*_3_, from which FA maps were calculated (Basser and Pierpaoli, 1996).

To assess reproducibility, images created in each of the four sessions needed to be coregistered to each other. We used three different methods for coregistration and compared their impact on measurement reproducibility.

1. A rigid body coregistration with 6 degrees of freedom (3 translations, 3 rotations and no scaling) was performed using SPM software (SPM5, http://www.fil.ion.ucl.ac.uk/spm/). This was done using a two pass procedure: to achieve a gross alignment of images, the first FA map of each subject was initially coregistered to a FA template in MNI space by a rigid body transformation, preserving each subject's individual anatomy. Then all four FA images were coregistered to this template aligned image, the average FA was calculated and the rigid body coregistration was repeated, using the average FA as target image. Coregistered images were resampled to 1 mm isotropic voxels using 2nd degree spline interpolation. This procedure will be referred to as ‘affine’ coregistration.
2. The same procedure was then repeated, including nonlinear warping (32 nonlinear iterations) for normalization to each subject's mean FA image. For the nonlinear normalization the subject's smoothed average FA image was used as a weighting mask, assigning more importance to regions with high FA for the normalization procedure.
3. We used FSL's tract based spatial statistics (TBSS) tools to normalize each single FA image to the provided FMRIB58_FA template image in MNI space. TBSS default settings were used for this nonlinear transformation.

The masks created by TBSS for each scan were combined to create an average mask image for each subject that was eroded by two voxels to exclude non-brain voxels for all further processing and analyses. For voxel wise comparison, the realigned FA images were smoothed with a 4 mm FWHM kernel.

### Regions of interest

**Fig. 1:**
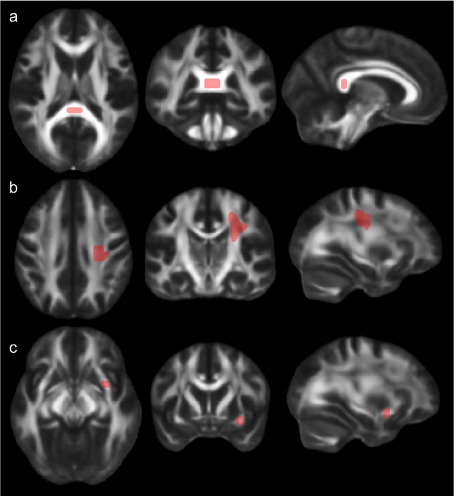
ROI placement in template space. a) Splenium of corpus callosum (SCC), b) left frontal white matter (LFWM) and c) left uncinate fascicle (LUF).

We chose three commonly used regions of interest (ROI), representatively reflecting different characteristics of white matter, and defined these ROIs manually on each subject's individual mean FA image in native space as well as on a FA template image in MNI space using MRIcro software (http://www.sph.sc.edu/comd/rorden/mricro.html) (Rorden and Brett, 2000) and the following anatomic guidelines:

1. A region representing an area of white matter with mainly parallel, densely packed fibers was defined in the splenium of the corpus callosum (SCC). A ROI of 0.8 cm^3^ was drawn in adjacent coronal slices, and the shape of the ROI was checked in sagittal slices (see Fig. 1a). To minimize partial volume effects at the edge of anatomic structures, ROI were restricted to the centre of the CC and 2 mm distance was kept to its anatomic boundaries.
2. A large, 3.5 cm^3^, region representing white matter with fibers of different and crossing orientations, was drawn in the left frontal white matter (LFWM), lateral to the commissural fibers from the CC and including the superior part of the corona radiata (Fig. 1b).
3. For the left uncinate fascicle (LUF), a smaller tract with lower average FA, a small 0.3 cm^3^ ROI was drawn in sagittal FA slices, selecting the first voxels with high FA values, ascending anteriorly from the inferior longitudinal bundle when scrolling from lateral to mesial. The anterior part of the core of the LUF was best defined in coronal slices where it can easily be depicted as a bright fiber bundle at the inferior frontal lobe (Fig. 1c).

All ROI were smoothed with a 3 × 3 × 3 voxel mean filter after drawing. ROI defined in template spaced were also backnormalized to each subject's individual native space and measurements were performed in both, template and native space.

For comparison with other studies that used all brain voxels or histogram based statistics to assess DTI reproducibility, we also determined statistics for a whole brain ROI, using each subject's thresholded *b*0 image to mask out CSF.

### Tractography

Probabilistic tractography was performed with FSL's probtrack algorithm, using the default settings with 5000 iterations per seed voxel. The abovementioned ROI were defined in template space, backnormalized to each subject's four individual scans and used as seed regions with the following constraints:

1. For the SCC ROI no further restrictions were made, the resulting tract mainly showing the commissural connections between homologous areas of the two parietal and occipital lobes.
2. For the LFWM ROI a waypoint mask in the brainstem was defined in the lowest axial slice, the resulting tract therefore showing the descending fibers of the corticospinal tract.
3. To track from the LUF ROI, exclusion masks were used in the sagittal midline to avoid crossing fibers and posterior to the vertex of the uncinate fascicle to exclude the inferior longitudinal bundle.

Tracking was performed independently for all four scans from each subject and the average FA within the tract and tract volume were calculated, thresholding the probability maps at 2%.

### Reproducibility maps

To assess the spatial distribution of FA reproducibility within the scan volume, reproducibility maps were generated. For each subject, a difference image was created for each scan, calculating the absolute (positive or negative) difference of each single FA voxel from the subject's average FA. An average absolute difference image was created as well as an average relative difference image, dividing the absolute difference by the average FA, thereby showing the percentage change of the initial FA value. All maps were normalized to MNI space to create the group average reproducibility maps.

### Statistics

ROI were applied to all four FA maps for each subject and ROI statistics were determined using FSLstats (FSL 4). Mean, standard deviation, minimum and maximum FA were extracted per ROI and analyzed with SPSS 14 (SPSS Inc., Chicago, IL, USA) and Microsoft Excel. For voxel wise comparison, AFNI (http://afni.nimh.nih.gov/afni) was used to extract individual voxel values from the SCC ROI for further correlation analysis.

The coefficient of variation (CV) is defined as the ratio of the measurements standard deviation *σ* divided by the mean *μ* and multiplied by 100. It allows an intuitive estimate of measurement variance expressed as relative percentage, regardless of the absolute measurement value. In previous studies on DTI test–retest reliability, the CV is the most commonly reported statistical measure. However, there are different ways to determine the CV for a given ROI:

• CV of the mean (CV_mn_): the mean value from each ROI is determined for each scan and the difference between these mean values is determined.
• CV of the median (CV_md_): instead of calculating the mean value from a ROI, the median value is determined and compared across scans. Assuming a symmetric distribution of values within a ROI, this should be close to the CV_mn_.
• CV of voxel wise comparison (CV_vw_): within each ROI, corresponding voxels from different scans are compared against each other and the CV_vw_ is determined for voxel wise differences.

CV_mn_ were calculated for each ROI and pairs of scans (intra-site and inter-site) per subject and for the group. CV_vw_ were calculated only for the SCC ROI, derived from both the raw and smoothed FA maps.

A different assessment of a method's reliability is the intraclass correlation coefficient (ICC) which relates the within-subject variation to the between-subject variation:

$$\text{ICC}=\frac{\sigma^{2}{}_{\mathrm{bs}}}{\sigma^{2}{}_{\mathrm{bs}}+\sigma^{2}{}_{\mathrm{ws}}}$$

where *σ*_bs_ = between-subjects standard deviation of the population and *σ*_ws_ = within-subject standard deviation for repeated measurements. The ICC expresses the fraction of the total variance in the data that is caused by true biological variation between subjects rather than by measurement error within subjects. For test–retest data of healthy controls, acquired under similar conditions, true within-subject differences will be small, and the method yielding the highest ICC will be preferable.

## Results

**Fig. 2:**
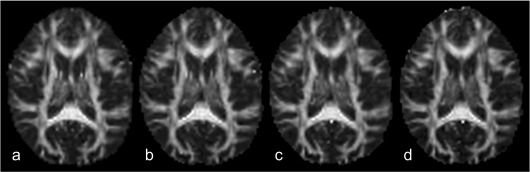
Reproducibility. The same mid-axial slice of the first subject's realigned FA maps from all four acquisitions is shown: a) site 1 scan 1, b) site 1 scan 2, c) site 2 scan 1 and d) site 2 scan 2. Note the details of gyral anatomy.

Visual inspection showed a very high similarity between the generated FA maps. Fig. 2 shows the same mid-axial slice from the four different scans of subject one. Detailed gyral anatomy was reliably reproduced.

### ROI characteristics

The cross subject mean FA, within-subject SD and between-subject SD are summarized in Table 2. The average within-subject SD across the four different scans was always lower than the between-subject SD for all FA measures. The between-subject CV_mn_ ranged from 3.1% to 12.1%.

### Coefficient of variation, CV

**Fig. 3:**
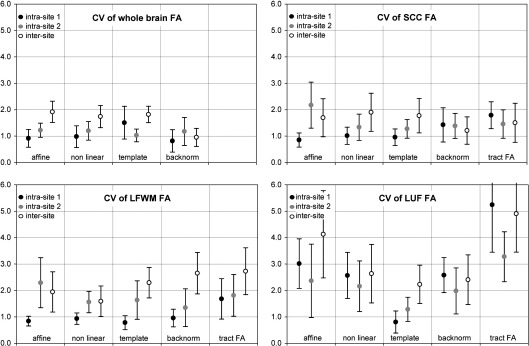
Coefficients of variation (CV, mean and SD from nine subjects). The plots show the results achieved with different image coregistration strategies for the four examined region: the first block in each plot shows results from the rigid body affine coregistration in subject's native space. The second block shows results from nonlinear warping to each subjects mean FA image in native space. The third block shows results from images normalized to template space, with all measurements done in template space. The fourth block shows results from ROI defined in template space and backnormalized to each subject's individual native space. For the three circumscribed regions, a fifth block is included, showing the CV for average FA values within the probabilistic tract seeded from that region.

CV_mn_ for intra- and inter-site rescans are summarized in Fig. 3.

Comparing the examined regions, the highest CV was found for the LUF, the smallest of the three regions, and therefore most prone to partial volume effects from imperfect coregistration and interpolation. Unsurprisingly the whole brain average FA showed the lowest variation and also the least dependence on the applied coregistration method.

Comparing the different coregistration methods, in general, affine coregistration resulted in bigger variation compared to any of the nonlinear methods for most measurements. For all three regions, the CV of FA within the tract was higher than the CV of the corresponding backnormalized seed region, on average by 50%.

**Fig. 4:**
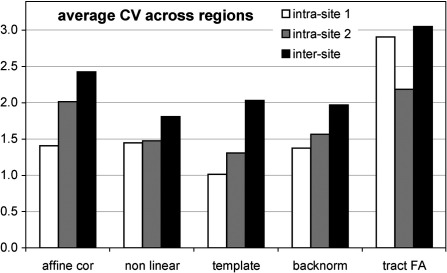
Average coefficients of variation (CV) across regions. Note the additional variation, introduced by probabilistic tracking, compared to the backnormalized seed regions. Affine coregistration performed worse than any of the three methods including nonlinear normalization steps (nonlinear in native space, template based and backnormalized from template).

Fig. 4 shows the average CV across all regions for a given coregistration method. The three nonlinear methods did not differ significantly, but affine coregistration performed worse than any of the three methods including nonlinear normalization steps (nonlinear in native space, template based and backnormalized from template). The average CV from these three methods were 1.3% for intra-site 1, 1.4% for intra-site 2 and 1.9% for inter-site scan–rescan.

There was a non-significant trend toward a higher intra-site CV for site 2 and both intra-site CV were significantly lower than inter-site CV (paired *T*-test, *p* = 0.0026 and *p* = 0.0015). However, using nonlinear coregistration, the average inter-site CV across regions still remained very low at 1.9%.

CV for the tract volume from the three tracts is not shown in the plots; the average was 8.4% for intra-site 1, 6.2% for intra-site 2 and 7.4% for inter-site—more than 2.5 times the variation than for the average FA within tract.

### Intraclass correlation coefficient, ICC

**Fig. 5:**
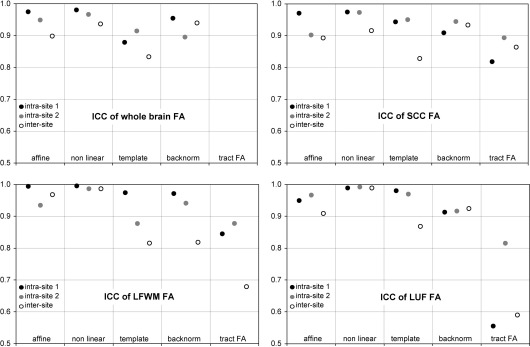
Intraclass correlation (ICC). Analog to Fig. 3 for CV. Note the lower ICC for template and backnormalized measures, compared to the first two methods in a subject's native space. This is mainly caused by a reduced between-subject variation for the template based methods (compare Table 2).

The ICC relates the within-subject variation to the between-subject variation. Results are plotted in Fig. 5 for all regions and methods. The ICC values were higher for the two normalization methods in native space (affine and nonlinear) compared to the two template based methods (template and backnormalized).

**Fig. 6:**
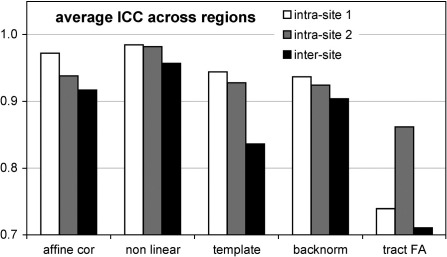
Average intraclass correlation (ICC) across regions. ICC was lower for template based methods (template and backnormalized) than in native space (affine coregistration and nonlinear). Tract FA showed much lower ICC than ROI analysis.

Like the CV values, ICC of all tract FA measures showed a much lower reproducibility than the corresponding ROI analyses (Fig. 6). The lowest ICC was observed for the LUF tract FA which was only 0.55 for intra-site 1 scan–rescan, compared to 0.91 for the corresponding ROI analysis.

### Voxel wise comparison

For the SCC, FA maps from the four scans were compared on a voxel by voxel basis. CV derived from voxel wise comparison (CV_vw_) of raw FA images were 4.2% for intra-site-1, 4.4% for intra-site-2 and 4.3% for inter-site, more than twice as big as those derived from the ROI mean value (CV_mn_). This illustrates noise in unsmoothed data at a single voxel level and also the averaging effect of a ROI analysis. However smoothing the FA maps with a 4 mm FWHM kernel before comparison reduced the CV_vw_ to 1.5%, 1.8% and 2.2% respectively, much closer to the ROI derived CV_mn_.

### Scanner differences

We found a consistent inter-site bias, FA values on site 2 were 1.0–1.5% lower than on site 1. This difference was slightly higher in areas with higher FA. Correction for this bias with a global scaling factor reduced the average inter-site CV for the nonlinear methods from 1.9 to 1.6%. This was no longer significantly different from the intra-site CV of 1.3% and 1.4% (paired *T*-test, *p* = 0.07 and *p* = 0.18).

### Reproducibility maps

**Fig. 7:**
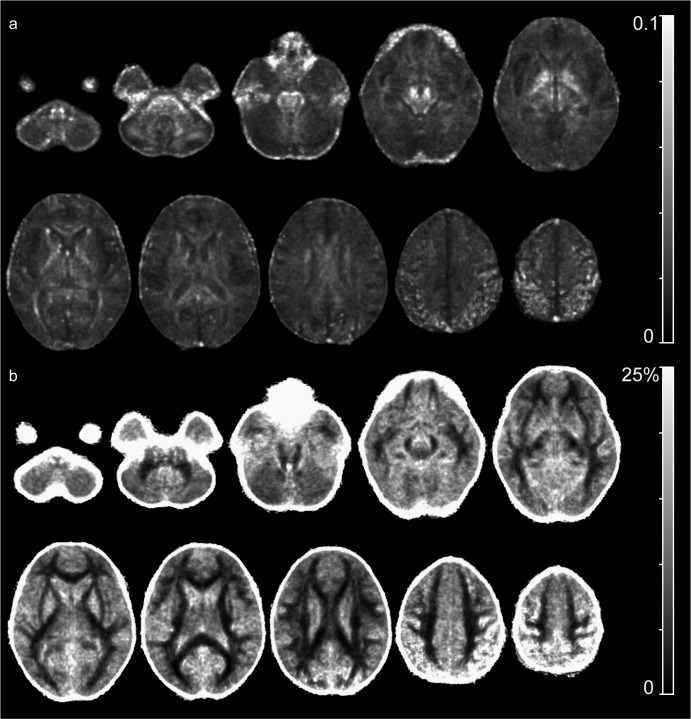
Regional distribution of FA reproducibility throughout the scan volume. a): Average absolute changes of FA values per voxel were highest close to skull base and around the brainstem. b): Average relative change, expressed as percentage change of the regional FA value stayed low in major white matter tracts but reached 10–15% in gray matter.

Assessing the regional distribution of FA reproducibility throughout the scan volume identified regions with less good reproducibility. (Fig. 7) The average absolute changes of FA values per voxel reached about 0.1 in the superior parietal lobe and around the brainstem. (Fig. 7a) The map showing the average relative change, expressed as percentage change of the regional FA value, resembles an inverse FA image with low changes in the major white matter tracts, staying well below 5%. (Fig. 7b) However, the map also shows that the average changes in cortical and subcortical gray matter were between 10 and 15%, reaching up to 25% in the superior parietal lobe.

## Discussion

We report for the first time at 3.0 T both intra-site and inter-site scan–rescan reproducibility of fractional anisotropy (FA) measures from DTI in nine healthy volunteers using identical scanners and acquisition protocols on two different sites. This is also the first study to assess the contribution of several image processing steps to overall reproducibility. Using appropriate coregistration techniques, intra-site and inter-site reproducibility of FA measures from a typical current best practice DTI protocol showed coefficients of variation (CV) below 2%.

### Intra-site comparison—ROI

Our intra-site CV values ranging from 0.8% to 3.0% were considerably lower than previously reported data obtained at either 1.5 T or 3 T, underlining the importance of factors other than field strength alone.

There are only two previous studies assessing rescan reliability of FA measures at 3 T. Bisdas et al. (2008) reported a CV of 2% for the SCC, only slightly larger than our average intra-site CV of 1.5% for manually drawn ROI. Their ROI in the SCC was similarly sized and two acquisitions were averaged with 16 diffusion weighted volumes each, resulting in a total number of 32 volumes, comparable to our protocol.

The second 3 T test–retest study, however, reported considerably larger CV_md_ of 3.0% and CV_vw_ of 6.5% for the whole cerebrum FA (Jansen et al., 2007) compared to our average intra-site CV_mn_ of 1.3% measured in template space. This is interesting, as the ‘whole cerebrum’ is the largest possible ROI and one would expect a low variation, simply because of the averaging effect of the large number of voxels. Indeed in our study the whole brain measures were most robust. For all four coregistration methods, the intra-site whole brain CV_mn_ stayed below 1.5%. The lower CV in our study may be due to the use of cardiac gating, as well as to the higher number of diffusion weighted directions—32 compared to the 15 used in Jansen's study—probably resulting in a higher signal to noise ratio (SNR) and better reproducibility in our data.

The majority of DTI reproducibility studies have so far been carried out on 1.5 T scanners. Heiervang et al. found a whole brain white matter CV of 0.78%, compared to our 1.3% for the whole brain, including gray and white matter (Heiervang et al., 2006). We did not segment images into gray and white matter, but our reproducibility maps have shown an approximately three times higher variation of FA in regions of gray matter and in gyri in which DTI voxels will include both gray and white matter, than in white matter, explaining the difference between these two whole brain measures. However, Heiervang's study reported a considerably larger regional CV of 4.81% for the CC, compared to our 1.1% in template space. The difference between whole brain and regional CV most likely reflects the different sized ROI volume: a small ROI is more prone to noise and partial volume effects and more likely to show a greater variation.

For all regions tested, our 3 T data showed consistently lower CV than previously reported from studies using 1.5 T scanners. Pfefferbaum et al. (2003) and Bonekamp et al. (2007) reported intra-site rescan CV for the CC, of 1.9% and 2.6% respectively, using a larger ROI than Heiervang's study. These values are still higher, but closer to our finding of 1.5% CV for affine coregistration. Increasing ROI size improves reproducibility, as long as contamination from surrounding structures with markedly different voxel values is avoided. This is especially relevant for the CC, where FA drops dramatically from ∼ 0.8 to essentially 0 in the surrounding CSF. Bonekamp et al. (2007) also assessed a ROI in the ‘superior corona radiata’, an area quite similar to our LFWM, and reported a CV of 3.8%, more than double our CV of 1.6%.

### Intra-site comparison—tractography

The additional variation introduced by a probabilistic tracking algorithm varies considerably. Two other studies have also assessed reproducibility of probabilistic tractography. Heiervang et al. (2006) investigated the reproducibility of tracking from a seed region in the corpus callosum and their reported CV of 1.94% is very similar to our 1.6% for the average FA within the tract. Comparable to our results, they reported a much higher variation of the tract volume with a CV of 5.03%, also very similar to our average intra-site CV of 4.9%. Reproducibility of callosal fiber tracking was also assessed by Ciccarelli et al. (2003). They reported a CV of 6.2% for the mean tract FA and 7.8% for the tract volume, both much higher than in our study (1.6% and 4.9% respectively) or in Heiervang's data. This is surprising, because this was the only other study including cardiac gating for the DTI acquisition, and this is expected to improve SNR and thereby aid good reproducibility. The high variation may stem from the specific tractography algorithm used in Ciccarelli's study.

### Inter-site comparison

No study on 3 T, and only very few studies on 1.5 T instruments have addressed inter-site reliability of DTI measures, with inter-site differences being consistently larger than intra-site measures, as expected. Pfefferbaum et al. (2003) reported higher variability between different scanners than for intra-site rescans, both for all supratentorial brain voxels (inter-site CV 1.93% versus intra-site 1.36%) and for a single ROI at the CC (5.2% versus 1.90%). The inter-site whole brain CV of 1.93% was similar to our 1.9% for affine coregistration, probably reflecting that a very large sample size partially compensates some regional differences in images from different scanners, even at 1.5 T. However, for the region of the CC, their inter-site CV was markedly larger (5.2%) than ours (1.7%).

Cercignani et al. also assessed inter-site and intra-site variability of histogram based DTI measures in eight and four healthy subjects respectively, scanned on two different 1.5 T systems with three different acquisitions (Cercignani et al., 2003). They proposed whole image histogram based measures rather than ROI-based measures. Using different scanners, CV of the whole brain histogram derived mean FA was significantly greater (7.71%) than different acquisition schemes on the same scanner (5.45%). Both CV were relatively high, compared to our results and other studies. Due to the small number of subjects for intra-site rescanning, no direct comparison of intra-site versus inter-site rescanning was made and intra-site rescanning variation was not reported in detail.

### Intraclass correlation coefficient, ICC

Comparing the different methods to define and align ROI, there was little difference between the CV achieved with the three methods including nonlinear transformations. Because of differences in the between-subject SD *σ*_bs_, the ICC depends more on the processing method. Defining ROI in each subject's native space measures values from a customized region, defined by every subject's individual anatomy. It is optimal to pick up between-subject differences and results in a bigger *σ*_bs_. Consequently the within-subject variation *σ*_ws_ between repeated scans contributes less, resulting in relatively high ICC values. Compared to the affine coregistration, the nonlinear normalization of images in native space minimizes the *σ*_ws_ by a better alignment and therefore achieves the highest ICC scores of all methods. The template based methods on the other hand reduce the *σ*_bs_ by normalizing the region and its measurement values across subjects, thereby decreasing ICC values. This illustrates how much a statistical measure like the ICC depends on details of the image processing pipeline. For example the inter-site ICC for the LUF ROI was 0.87 for measurements in template space and 0.99 for measurements in individual ROI in native space after nonlinear normalization. Furthermore, this ‘equalizing’ effect of normalization to template space should to be kept in mind in patient studies, as it may indicate a loss of sensitivity to pathological changes.

Comparing our ICC values to previous studies, we also found a better reproducibility in our study. Jansen reported an ICC for whole brain median FA at 3 T of 0.73 (Jansen et al., 2007), where we measured intra-site ICCs of 0.88 and 0.91. In their 1.5 T study, Bonekamp et al. reported an ICC of 0.65 for a ROI in the corpus callosum (Bonekamp et al., 2007) which was 0.97 and 0.90 in our study.

### Scanner differences

**Fig. 8:**
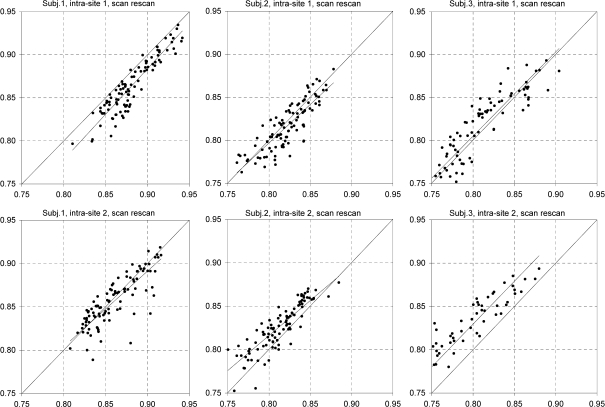
Sources of error in reproducibility. This figure shows scatterplots with trendlines comparing the 100 individual voxels from the SCC ROI, derived from the smoothed FA images of the first three subjects. The upper images show intra-site-1 scan–rescan correlations, the lower ones intra-site-2 correlations. The plots illustrate two effects contributing to different measurement values in repeated scans: subject 1 shows a shift of mean between the site 1 scans and single voxel outliers in the site 2 scans with an otherwise good correlation. Subjects 2 and 3 show an apparent shift of mean between scans at site 2.

The use of two nominally identical scanners and identical acquisition protocols minimized inter-site variability. However, in spite of identical hardware, firmware and software, and identical procedures, there were still slight differences between the two scanners and the average inter-site variation was about 40% higher than the intra-site variation in our setting. Even though this is still an improvement over using different scanners with scanner variation being typically twice as high as within one site (Pfefferbaum et al., 2003), these findings also show that nominally identical scanners may operate, and be operated, slightly differently, in varying conditions and should be assessed independently. This was also shown by the consistent inter-site bias, with slightly lower FA values on site 2 (1–1.5% difference). In case of a consistent bias, it may be feasible to apply a global scaling factor to improve cross-site comparability of measurements. In our study this has reduced the average inter-site CV to under 1.7%, which was not significantly different from the intra-site CV of site 2. In our study there was a trend towards lower variation between scans on site 1 than site 2. A possible explanation is the fact that the scanner is used more intensively on site 2, resulting in higher wear and more frequent servicing and calibrations. Such (re-)calibration may also contribute to the shifts of the mean seen in the offset of the trend lines in Fig. 8; because the *b*-value is proportional to the square of the applied gradient strength, even small changes in calibration may lead to relatively large changes in MD (and, if different along different gradient axes, FA). Frequent scanner servicing and calibration by the manufacturer is usually assumed to be beneficial, keeping the scanner performing optimally. However, it may have a disadvantageous effect on data reproducibility for DTI. Nagy et al. have demonstrated a method to calibrate gradients for DTI and this might further improve reproducibility (Nagy et al., 2007).

### Other factors influencing reproducibility

The degree of reproducibility achievable in any study is likely to be related to a number of factors. Scanner parameters like field strength or gradient performance, as well as acquisition parameters like voxel size, number of diffusion weighted directions and the use of cardiac gating, are all likely to play a role (Alexander et al., 2006; Ni et al., 2006), as are issues such as the protocol used for subject (re)positioning. The fact that nonlinear coregistration improved reproducibility compared to affine coregistration within each subject shows that different nonlinear distortions appear when scanning the same healthy subject on the same scanner. Most likely these differences are due to small changes in head positioning, but other biological factors such as fluid in paranasal sinuses may also play a role. The effect of various acquisition parameters on DTI data quality has been addressed in detail in several studies (Ardekani et al., 2006), mostly on 1.5 T scanners (Papadakis et al., 1999; Ni et al., 2006; Landman et al., 2007). Many of these studies used quite specific measures of the error or data quality which cannot easily be translated to a measure like the CV typically used to address data reproducibility.

Generalizing our results to other protocols is beyond the scope of the current study, and reproducibility of any proposed protocol will need to be assessed before starting large scale multi-centre studies. Our results do show, however, that with appropriate parameters, acceptable inter- and intra-site reproducibility can be achieved using a contemporary 3 T scanner and a 10 minute DTI acquisition protocol.

One factor which has a particular impact on DTI data quality, and should therefore be considered when setting up multi-site studies, is the use (or otherwise) of cardiac gating. DTI sequences are designed to detect molecular diffusion and thus are naturally very sensitive to motion. Pulsation related movement of the brain is therefore a significant source of noise in a DTI acquisition, particularly at the level of the brainstem. This can be reduced by limiting the acquisition time to diastole when pulsation effects are minimal, although the resulting gain in data quality is achieved at the expense of a prolonged acquisition time. Several studies have addressed the time efficiency of cardiac gating: Skare et al. found a 2.5–4 times higher variation in certain regions in ungated DWI images (Skare and Andersson, 2001), while Gui et al. reported that cardiac gating roughly halved the ‘total variance of the diffusion tensor’ (Gui et al., 2008). Recently, Chung et al. reported an almost threefold reduction of images with severe artifacts by cardiac gating, while the gating scheme increased scanning time by only 27%. They therefore state that for a given possible scanning time it is more efficient to use cardiac gating than to acquire more excitations (data averages) or more diffusion weighting directions (Chung et al., 2009). Our gating scheme used a minimum delay after peripheral gating and allowed the acquisition of two to four slices per RR cycle, depending on the subject's heart rate.

### Reproducibility versus sensitivity

Test–retest studies allow for an estimation of reproducibility, i.e. within-subject differences. Reproducibility, however, represents only one aspect of a measurement. A method can conceivably be very reproducible at the expense of not reflecting parameters of interest at all.

The between-subject CV_mn_ differs significantly between the four ROI. The much higher between-subject CV_mn_ for the LFWM and LUF FA results in relatively higher ICC values, as the same amount of ‘noise’ between repeated scans is less relevant relative to the larger true biological variation between subjects.

These differences illustrate the potential discrepancies between various statistical approaches to assess the test–retest reliability and their suitability for a given question. For example the ICC might not be an ideal measure in healthy control populations, as the relatively low between-subjects variation may be unrepresentative for a patient population where different degrees of pathologies will result in higher between-subjects variation. The CV may not be an ideal measure of precision, because of its dependency on the measured mean value. Furthermore, neither the CV nor the ICC takes into account possible shifts of the mean value (i.e. accuracy), which has to be assessed independently. A method's reproducibility needs to be balanced against its sensitivity (Heiervang et al., 2006). Measures with a very good reproducibility—such as whole brain measurements—might be insensitive to pathological changes in clinical studies. On the other hand, clinically more relevant, hypothesis driven measures, targeted to a specific, smaller region of interest, may show greater sensitivity to pathological changes—and at the same time have a poorer reproducibility, due to a higher sensitivity to subtle variations in data or influences from image processing. It is therefore crucial to know the margin of reproducibility for a given measure, to detect clinically relevant changes beyond the method's noise.

## Conclusion

Using the methods described, with two identical 3 T scanners, we achieved a consistently low variation of FA measures between scans for both intra- and inter-site rescanning with average CV between 1% and 2%. Compared to previous studies on 1.5 T scanners, this represents an improvement of reproducibility by approximately a factor of two.

Improvements in MRI data reproducibility are the result of a number of contributing factors. The gradient performance of a contemporary 3 T scanner allows shorter echo times for a particular degree of diffusion sensitization (*b*-value), and this, along with increased field strength, increases SNR. The use of cardiac gating helps to reduce pulsation related motion artifacts in the inferior part of the brain, and, the use of cardiac gating has been shown to be time efficient and is usually recommended in present-day DTI. For a ROI-based study, careful positioning of the ROI can aid reproducibility by reducing partial volume contamination from areas of high variability. Using nonlinear normalization between scans is beneficial to account for different distortions between scans. Probabilistic tractography introduced approximately 50% additional variation compared to a ROI analyses. This should be justified by a clear anatomical hypothesis about the involvement of a specific white matter tract, when tracking is used rather than a ROI analysis. Tract volume showed the lowest reproducibility with an average CV of more than 7%. Reproducibility of FA in subcortical gray matter and cortical gyri containing white and gray matter within the scale of DTI voxels was poor, with variations up to 15%, illustrating that DTI is more robust for assessing white matter characteristics.

The observed inter-scanner differences illustrate that nominally identical scanners give slightly different results. However, given the fact that cross-site variation between different scanners is usually more than double the intra-site reproducibility, and that the overall variation in our study was much lower than previously reported, our findings support the feasibility of cross-site pooling of DTI data from identical scanners. An average inter-site CV of less than 2% for FA measures is encouraging, and paves the way for multi-centre studies of DTI, allowing the recruitment of larger subject numbers across different sites.

## Figures and Tables

**Table 1:** Comparison of results with previous studies on DTI test–retest reliability. The values shown for this study are the average CV from the three nonlinear methods. See Fig. 3 for other values. dwd = diffusion weighted directions, CV = coefficient of variation in %, WSV = within-subject variation, cg = cardiac gating, inter-site measures are printed italic.

Study	Field strength	Acquisition	Subjects	Repeated scans/measures	Statistics used	Reported CV whole brain [%]	Reported CV corpus callosum [%]	Reported CV other regions [%]	Comment
Scanner	dwd, repetitions voxel size *x*, *y*, *z* duration	Age mean ± SD or [range]
This study	3.0 T GE Signa + GE Signa	32 dwd 2.4 × 2.4 × 2.4 mm 10 min	9 volunteers 34 ± 8	Intra-site rescan ×2 Inter-site rescan ×2	Mean FA from ROI	1.1 *1.5*	1.2 *1.6*	LFWM 1.2 *LFWM2.2*	ROI SCC: 0.8 cm^3^ LFWM: left frontal white matter
									
Bisdas	3.0 T Philips Intera	16 dwd × 2 2 × 2 × 3 mm	12 volunteers 38 ± 11	Intra-site rescan ×2	Mean FA from ROI		2		ROI SCC: 0.2 cm^2^
									
Jansen	3.0 T Philips Achieva	15 dwd 2 × 2 × 2 mm 10 min	10 volunteers 26 ± 2	Intra-site rescan ×2	Median FA Voxel wise	3.0 6.5			Images normalized to MNI Smoothed 6 mm FWHM
									
Bonekamp	1.5 T GE	15 dwd × 2 2.5 × 2.5 × 5 mm 5 min	10 volunteers 14.1 ± 2.8	Intra-site rescan ×2	Mean FA from ROI		2.6	SCR 3.8	SCR: superior corona radiata ROI: 16 voxels in single slice
									
Heiervang	1.5 T Siemens Sonata	60 dwd 2 × 2 × 2 mm	8 volunteers [21–34]	Intra-site rescan ×3	Variable	0.78 (mean FA from white matter)	4.81		Images normalized to MNI ROI in GCC, size 9 voxels
									
Ciccarelli	1.5 T GE Signa	60 dwd 2.5 × 2.5 × 2.5 mm 20–30 min (cg)	10 volunteers 37.5 ± 9.7	Intra-site rescan	Mean FA in tract		6.2		4 subjects rescanned ROI: ‘callosal fibers’ after tracking
									
Cercignani	1.5 T Philips Gyroscan + Siemens Vision	6 dwd ×10 or 8 dwd ×8 1.95 × 1.95 × 5 mm	12 volunteers 28.9 [23–33]	Intra-site rescan ×2 Inter-sequence rescan ×3 Inter-site rescan ×2	Mean FA from histogram	Not reported 5.45 *7.71*			4 subjects rescanned intra-site 8 subjects rescanned inter-site
									
Pfefferbaum	1.5 T GE Echospeed + GE Twinspeed	6 dwd ×6 Not reported	10 volunteers [21–33]	Intra-site rescan ×2 Inter-site rescan ×2	Mean FA from ROI	1.36 *1.93*	1.90 *5.20*		Images coregistered to common space ROI: ‘outlined in midsagittal FA’

**Table 2:** Group characteristics of the examined regions: ROI size, group mean FA. Average within (SD_ws_) and between (SD_bs_) subjects SD is shown for all four analysis methods.

Region	ROI size [cm^3^]	Mean FA	Affine		Nonlinear		Template		Backnormalized	
SD_ws_	SD_bs_	SD_ws_	SD_bs_	SD_ws_	SD_bs_	SD_ws_	SD_bs_
Whole brain	–	0.28	0.0038	0.0145	0.0033	0.0157	0.0034	0.0073	0.0031	0.0087
SCC	0.8	0.84	0.0148	0.0456	0.0118	0.0550	0.0105	0.0368	0.0117	0.0388
LFWM	3.5	0.48	0.0095	0.0499	0.0074	0.0613	0.0080	0.0227	0.0088	0.0273
LUF	0.3	0.39	0.0169	0.0621	0.0102	0.1035	0.0055	0.0219	0.0094	0.0270

SCC: splenium of corpus callosum, LFWM: left frontal white matter, LUF: left uncinate fascicle.
